# Event-Related Potentials and Emotion Processing in Child Psychopathology

**DOI:** 10.3389/fpsyg.2016.00564

**Published:** 2016-04-29

**Authors:** Georgia Chronaki

**Affiliations:** ^1^Developmental Cognitive Neuroscience Laboratory, School of Psychology, University of Central LancashirePreston, UK; ^2^School of Psychological Sciences, University of ManchesterManchester, UK

**Keywords:** ERPs, emotion, children, adolescents, psychopathology

## Abstract

In recent years there has been increasing interest in the neural mechanisms underlying altered emotional processes in children and adolescents with psychopathology. This review provides a brief overview of the most up-to-date findings in the field of event-related potentials (ERPs) to facial and vocal emotional expressions in the most common child psychopathological conditions. In regards to externalizing behavior (i.e., ADHD, CD), ERP studies show enhanced early components to anger, reflecting enhanced sensory processing, followed by reductions in later components to anger, reflecting reduced cognitive-evaluative processing. In regards to internalizing behavior, research supports models of increased processing of threat stimuli especially at later more elaborate and effortful stages. Finally, in autism spectrum disorders abnormalities have been observed at early visual-perceptual stages of processing. An affective neuroscience framework for understanding child psychopathology can be valuable in elucidating underlying mechanisms and inform preventive intervention.

## Introduction

The worldwide prevalence of mental disorders in children and adolescents is about 13% and continues to rise (Polanczyk et al., 2015). As the majority of adult mental health disorders begin in childhood and adolescence, it is important to gain a better understanding of the causal mechanisms as well as the factors reducing risk and increasing resilience in the young to help develop effective prevention strategies. In the recent years, there has been a renewed interest in emotion dysregulation as a mechanism increasing the risk for a range of psychopathological conditions (Kret and Ploeger, 2015). Understanding the neurobiology of emotion processing in child psychopathology can advance knowledge of underlying mechanisms and aid the identification of intervention targets (Pine, 2007).

Understanding other’s emotions is critical in social interaction. Theoretical debates have focused on whether brain structures are specialized for processing social information or whether social cognition is part of general cognitive processes applied to social behavior (Adolphs, 2009). Empirical research has supported the proposal that there is a network of specific brain areas preferentially involved in the processing of social information, a network often referred to as the ‘social brain’ (Brothers, 1990; Johnson et al., 2005; Adolphs, 2009). Developmental psychology has demonstrated that the ability to understand other’s feelings and mental states develops in the first 4 years of life (Frith and Frith, 2003). Developmental neuroscience frameworks can be valuable for the study of emotion processing. Development provides a unique opportunity to study the neural correlates of emotion processing as they emerge at different ages (De Haan et al., 2003; Grossmann et al., 2007). This approach can provide answers to the question of ‘when’ the developing brain begins to become ‘tuned’ to its social environment. Event-related potentials (ERPs) represent a useful, non-invasive methodology to understand the timing (in a millisecond resolution) of the sensory, perceptual, and cognitive processes underlying social information processing (Nelson and Luciana, 2001). As neural substrates implicated in social processing become more specialized over development (Johnson et al., 2009), ERPs can inform our understanding of whether neurally separate components have the potential to be specialized for processing emotional information (De Haan and Gunnar, 2009). Finally, ERP methods are useful in conceptualizing not only typical but also atypical development as they can reveal individual differences which may not be evident in observable behavior. Developmental transitions in particular, such as early childhood and adolescence, represent important landmarks in mental health trajectories and are accompanied with a unique set of opportunities and challenges (Blakemore, 2010) which overlap with important neurobiological changes in emotion processing.

This mini-review aims to briefly summarize the ERP components implicated in facial and vocal emotion recognition in typical and atypical development. For this mini-review, computerized searches of articles published until 2015 were conducted using the PubMed, Psycinfo, Science Direct and Nature journals online databases. The following terms ERPs, facial, vocal, emotion recognition, child, adolescent, psychopathology, externalizing, internalizing, ADHD, CD, ASD, anxiety, depression, were entered into the databases. In addition, the table of contents of journals that often publish articles relevant to this topic were reviewed including Journal of Child Psychology and Psychiatry, Frontiers in Neuroscience, Human Brain Mapping, Biological Psychiatry, Nature Neuroscience, Developmental Science, Social Neuroscience and American Journal of Psychiatry. Finally, the reference lists of relevant articles were scanned for pertinent studies. Only studies written in English were included (see **Table 1**).

**Table 1:** A summary of empirical findings of altered ERP responses to facial and vocal emotional stimuli in children and adolescents with psychopathology.

Psychopathology type	*n*	Age (Years)	Sample	Task	Emotion	ERP effect
**ADHD**						
**Facial cues**						
Williams et al., 2008	51 ADHD 51 controls	8–17	Clinical	Emotion recognition	A, H, S, F, Di, N	↓ P120, ↑ N170, ↓ P300 amplitudes to anger in ADHD
Chronaki et al., 2010	41 children	6–11	Community	Emotion recognition	A, H, N	↓ Slow Wave to anger with increased hyperactivity
Tye et al., 2014	18 ADHD 26 controls	8–13	Clinical	Emotion recognition	A, H, F, Di, N	Reduced fear and happy N400 modulation in ADHD
Köchel et al., 2014	16 ADHD 16 controls	8–12	Clinical	Emotional Go/NoGo	A, H, S, N	↓ P300 amplitude in ADHD
**Vocal cues**						
Chronaki et al., 2015a	25 ADHD 25 controls	6–11	Clinical	Emotion recognition	A, H, N	↑ N100 amplitude to anger in ADHD
**Conduct disorder**						
**Vocal cues**						
Hung et al., 2013	20 CD 20 controls	13–19	High-secure offenders	Oddball Neutral-‘standards’ Fear/sad-‘deviants’	F, S, N	↑ MMN amplitude to fear in CD
**Anxiety and depression**						
**Facial cues**						
DeCicco et al., 2012	32 children	5–7	Community	Reappraisal	Pleasant, unpleasant, N	↑ LPP amplitude to unpleasant in high anxiety
Solomon et al., 2012	39 children	5–7	Community	Passive viewing	Pleasant, unpleasant, N	↑ LPP amplitude to unpleasant in fearful Children
Kujawa et al., 2015	53 Anxiety 37 controls	7–19	Clinical	Emotional face-matching	A, H, F, N	↑ LPP amplitude to anger and fear in anxiety
**Autism spectrum disorder**						
**Facial cues**						
Dawson et al., 2004	29 ASD 22 controls	3–4	Clinical	Emotion recognition	F, N	No emotion N300 and NSW modulation in ASD
Batty et al., 2011	15 ASD 15 controls	5–16	Clinical	Implicit emotion processing	A, H, S, F, Di, Sur, N	↑ P1 and N170 latency across emotions in ASD
Wagner et al., 2013	18 ASD 20 controls	13–21	Community (with ASD diagnosis)	Passive viewing	A, F, N	No emotion P1 and N170 modulation in ASD
Apicella et al., 2013	10 ASD 12 controls	6–13	Clinical	Passive viewing	H, H, N	↓ P1 and N170 amplitude ↑ P1 and N170 latency in ASD
Tye et al., 2014	19 ASD 26 controls	8–13	Clinical	Emotion recognition	A, H, F, Di, N	↓ N170 amplitude across emotions in ASD
**Vocal cues**						
Chin-hsuan, 2011	23 ASD 23 controls	NA	Clinical	NA	A, H	↓ MMN amplitude to anger in ASD
Korpilahti et al., 2007	13 Asperger syndrome 13 controls	9–12	Clinical	Passive oddball Happy-‘standard’ Angry – ‘deviants’	A, H (tender)	↑ N100 and MMN latency across emotions in Asperger
**Multimodal**						
Lerner et al., 2013	34 ASD No controls	10–16	Clinical	Emotion recognition	A, H, S, F (faces and voices)	N100 and N170 latencies were positively correlated with emotion recognition errors in ASD


*A, anger; H, happy; S, sad; F, fear; Di, disgust; Sur, surprise; N, neutral. ERP effects relate to findings in the experimental group (i.e., ADHD).*

## Typical Development

Theoretical models for recognizing facial emotional expressions emphasize that conceptual knowledge of emotion signaled by the face is preceded by early perceptual processes by salient stimuli (Bruce and Young, 1986; Haxby et al., 2000). The N170 is an occipitotemporal potential traditionally linked to sensitivity in processing information from human faces (Bentin et al., 1996; Taylor et al., 1999). Some studies have shown that the N170 is sensitive to facial emotion in adults (Batty and Taylor, 2003; Blau et al., 2007), although other studies have not found facial emotion modulation of the N170 (Eimer and Holmes, 2002; Herrmann et al., 2002; Eimer et al., 2003). Infant research has identified the N290 as a developmental precursor to the adult N170 (Halit et al., 2003, 2004). Emotion effects on the N170 have been observed in older (14–15-years-old) compared to younger (4–12-years-old) children, with N170 amplitudes being larger for negative (anger, sad) compared to positive (happy) and neutral faces in emotion recognition tasks (Batty and Taylor, 2006). Compared to the N170 proposed to index ‘fine-grained’ sensitivity to facial emotion emerging during adolescence, a parietal–occipital P1 component (∼120 ms) has been suggested to reflect global and ‘superficial’ processing of facial emotion that is present in younger children (Batty and Taylor, 2006; Vlamings et al., 2010). Beyond early components, later components such as the late positive potential (LPP), a parietal–occipital component evident from around 300 ms, show sensitivity to the emotional content of human faces and are proposed to signify elaborative or effortful processing of emotionally significant stimuli in healthy adults (Hajcak et al., 2010). The LPP has been shown to be sensitive to facial emotion in children. In particular, the LPP was larger in amplitude to angry compared to happy faces in 7-year-old children in emotion recognition tasks (Kestenbaum and Nelson, 1992) and sad compared to neutral faces at occipital areas in 6-year-old children in a passive viewing paradigms (Kujawa et al., 2012).

Despite a number of studies using facial stimuli, considerably less is known about the neural development of vocal emotion processing. This is surprising given the prominent role of vocal emotional expressions in children’s social interactions. Brain potentials in response to voice compared to non-voice sounds emerge between 160 and 200 ms on frontocentral (positivity) and occipital (negativity) sites in healthy adults (Charest et al., 2009). This suggests that the neural processing of voices and faces (‘face-specific’ N170) occur at similar time points explaining the integration of such signals in real-life social interactions (Campanella and Belin, 2007). In healthy adults, the recognition of emotion from vocal signals (i.e., ‘prosody’) is represented in the brain by a series of ERP components. According to a three-process model of emotional prosodic-processing, a temporal N100 component is suggested to reflect early sensory processing of vocal expressions, followed by a P200 component, proposed to reflect integration of prosodic acoustic cues and finally, frontal late latency components (i.e., P300, N400) reflecting cognitive-evaluative judgments such as labeling emotional expressions (Schirmer and Kotz, 2006). In adults, vocal emotion effects have consistently been observed in the N400 component (Bostanov and Kotchoubey, 2004; Paulmann and Kotz, 2008). The human brain begins to become sensitive to vocal signals of emotion from the first months of life (review by Grossmann and Johnson, 2007). Despite a number of infant studies, very little is known about the neural development of vocal emotion processing in childhood. In typically developing 6–11-year-old children differential ERPs to distinct vocal expressions of emotion (angry, happy, and neutral) have been identified in an emotion recognition task (Chronaki et al., 2012). These consisted of an early, N100 (90–180 ms) and a later, N400 (380–500 ms) component observed in more posterior (parietal–occipital) regions compared to adults (Chronaki et al., 2012). Further research is needed in the neural development of vocal emotion processing in children and adolescents.

## Atypical Development

An emerging body the ERP literature supports the idea that sensory, perceptual, and cognitive processing stages of emotion recognition may be altered in children with psychopathology. The section that follows reviews some landmark studies in children with externalizing and internalizing problems and autism spectrum conditions.

## Attention-Deficit/Hyperactivity Disorder

Attention-deficit/hyperactivity disorder (ADHD) is the most common neurodevelopmental disorder characterized by developmentally inappropriate levels of inattention, hyperactivity, and impulsivity (Americal Psychiatric Association [APA], 2013). Motivational processes (Sonuga-Barke and Fairchild, 2012) are implicated in ADHD and emotion dysregulation is recognized as an important clinical feature of the condition (Shaw et al., 2014; Bunford et al., 2015).

Although, some theories suggest that emotion processing difficulties in children with ADHD may result from general inattention or impulsiveness, socio-cognitive models have argued in favor of emotion-specific difficulties (review by Uekermann et al., 2010). Behavioral studies have shown that individuals with ADHD present deficits in the recognition of emotions (especially negative emotions) from facial expressions (see Uekermann et al., 2010) and that these deficits can be independent of cognitive functions such as attention (Bisch et al., 2016) and performance in non-emotion tasks (Rapport et al., 2002). Emotion recognition deficits are associated with behavior problems already in preschool (Chronaki et al., 2015b) and school-aged children (Pelc et al., 2006; Yuill and Lyon, 2007). ERP correlates of these deficits have only recently been identified. Adolescents with ADHD have been shown to display reduced occipital P120, followed by increased N170 and reduced temporal P300 amplitudes to anger and fear in a facial emotion recognition task (Williams et al., 2008). These findings may suggest reduction in occipital activity during the early perceptual processing of anger (120 ms), followed by increased activity during structural encoding stages (∼170 ms) and later reduction in temporal activity reflecting context processing of anger (∼300 ms). Similarly, hyperactivity was negatively associated with occipital Slow Wave amplitudes to facial anger in an emotion recognition task in a community sample of 6–11-year-old children (Chronaki et al., 2010). Similar work has shown that impairments in response inhibition to angry faces have been associated with reduced P300 amplitudes in a Go/Nogo task in boys with ADHD compared to controls (Köchel et al., 2014).

The only ERP study to date using vocal stimuli has shown enhanced N100 and attenuated P300 amplitudes to vocal anger in 6–11-years-old with ADHD in an emotion recognition task using pure prosodic stimuli (Chronaki et al., 2015a). The N100 effect persisted after excluding children with comorbid Conduct Disorder. This pattern of results possibly reflects hyper-vigilance to vocal anger in ADHD at early and almost automatic processing stages consistent with an automatic and less controlled processing style in ADHD (Oades et al., 1996). These findings are consistent with near-infrared spectroscopy work showing stronger supramarginal gyrus activation to sentences with angry intonation in children with ADHD (Köchel et al., 2015) and functional magnetic resonance imaging (fMRI) work showing enhanced frontal and posterior cingulate cortex activation to anger from facial expressions in 10–17-years-old with ADHD compared to controls (Marsh et al., 2008). Results should be interpreted in the context of recent conceptual models of emotional dysregulation in ADHD involving a circuitry underpinning deficits in rapid early orienting to emotion (i.e., ventral striatum, amygdala; Shaw et al., 2014).

## Conduct Disorder

Conduct disorder (CD) is a condition at the severe end of a continuum of oppositional defiant behaviors (Americal Psychiatric Association [APA], 2013). The majority of studies in emotion processing in CD and associated conditions have employed behavioral and fMRI methods and have shown pervasive deficits in the recognition of a range of emotions from facial and vocal modalities (meta-analysis by Dawel et al., 2012). A recent ERP study has shown that young offenders with CD displayed stronger mismatch negativity (MMN) to fearful syllables in a passive listening task with no difference found in controls. This findings may reflect enhanced pre-attentive auditory change detection for distressful stimuli in youth with CD (Hung et al., 2013). Despite methodological differences, these results are generally inconsistent with evidence from behavioral (Blair et al., 2005; Dadds et al., 2008; Fairchild et al., 2009) and functional neuro-imaging (Jones et al., 2009) studies which show *reduced* sensitivity to fearful facial expressions in active-attention tasks. These findings should be considered in the context of theoretical frameworks suggesting that failure to inhibit antisocial behaviors may be the result of lower sensitivity to distress-related cues from others such as fear (Blair, 2001).

There is a striking lack of empirical studies on the temporal processing of emotion in youth with CD. Further research is necessary before drawing any conclusions. In addition, given the high rates of comorbidity between CD and ADHD, future research should examine the electrophysiological correlates of emotion processing in ADHD, ADHD+CD, and CD to clarify the role of common or distinct neural pathways.

## Anxiety and Depression

The experience of negative affect (i.e., anxiety and depression) in children and adolescents has been closely associated with emotion processing (Hadwin and Field, 2010). Behavioral work in this area has predominantly been guided by theoretical frameworks of attentional biases to threat (Bar-Haim et al., 2007). The ERP literature points to the direction of enhanced neural response to threat (i.e., anger) stimuli in anxious children, as reflected by larger amplitudes of the LPP component, proposed to reflect elaborative or effortful processing of emotional stimuli (Schupp et al., 2000; Hajcak et al., 2010). Recently, Kujawa et al. (2015) found that relative to healthy controls, 7–19-year-old diagnosed with social anxiety, separation anxiety, and generalized anxiety disorders showed enhanced LPP amplitudes to angry and fearful faces during an emotional face-matching task. This is consistent with earlier research using pictorial stimuli which has found increased processing of unpleasant compared to neutral pictures (reflected by the posterior LPP amplitudes) in a community sample of 5–7-year-old with high anxiety (DeCicco et al., 2012). Similar results have been found in 5–7-year-old children with inhibited and fearful behavior (Solomon et al., 2012). ERP research in emotion processing in childhood depression is more limited. In the study by Kujawa et al. (2015), higher depressive symptoms were associated with reduced LPP amplitudes to angry faces in 7–19-years-old diagnosed with an anxiety disorder (Kujawa et al., 2015). Results partly support adult studies linking depression to blunted or reduced emotional response (as reflected by the LPP), consistent with theories suggesting disengagement from emotional stimuli more generally in depression (Proudfit et al., 2015). In summary, preliminary findings support the LPP as a neural marker of neurobiological vulnerability to threat in childhood internalizing symptoms. Future work should aim to disentangle the role of anxiety and depression in the neural processing of threat and explore whether existing effects generalize to vocal modalities.

## Autism Spectrum Disorder

Autism spectrum disorder (ASD) refers to a range of conditions characterized by impairment in social interaction and communication (Americal Psychiatric Association [APA], 2013). Children with ASD find social stimuli less salient than non-social stimuli (Stavropoulos and Carver, 2014) and present difficulties in recognizing other people’s emotions (see meta-analysis by Uljarevic and Hamilton, 2013). However, not all studies have supported emotion processing deficits in ASD (Jones et al., 2011). Further, it is not clear from behavioral studies whether existing deficits are emotion-specific or whether they are secondary to domain-general processing abnormalities (i.e., attention, sensory-perceptual processing).

Event-related potentials research has partly supported an atypical pattern of facial emotion processing in ASD. Typically developing 3–4-years-old displayed larger N300 amplitudes to fearful than neutral faces, while children with ASD did not show this effect in a passive viewing task (Dawson et al., 2004). Similarly, the amplitude of the face-sensitive N170 component varied with emotional expression only in typically developing adolescents aged 13–21 but not in adolescents with ASD who showed reduced neural differentiation between angry, fearful, and neutral facial expressions in a passive viewing task (Wagner et al., 2013). In an implicit emotional task, 10-years-old children with autism displayed longer P100 and N170 latencies and smaller P100 amplitudes to facial expressions of emotion including anger, disgust, happiness, sadness, surprise and fear. In this study, only the P1 amplitude remained affected in autism, after children with autism were matched by verbal equivalent age to controls, suggesting abnormalities at early stages of rapid visual perceptual processing (Batty et al., 2011). These findings are consistent with a slowed neural speed of face processing (McPartland et al., 2004) already present at 3 years in ASD (Webb et al., 2006). Recent research has shown that relative to controls, 6–13 years-old with ASD presented delayed latencies and reduced amplitudes of early components (P100, N170) regardless of emotion type in an implicit face-perception task whereby children viewed fearful, happy, and neutral faces and were asked to press a button when a cartoon stimulus was presented (Apicella et al., 2013). Results are consistent with fMRI work showing no impairments in the cognitive labeling of basic facial emotions in adolescents with ASD (Wang et al., 2004). More recently, children with ASD and comorbid ADHD have been shown to display reduced N170 amplitude across a range of facial emotions and particularly for fearful compared to neutral expressions in an emotion discrimination task (Tye et al., 2014), confirming work showing abnormalities at an early structural encoding processing stage.

Few studies have investigated the neural processing of vocal emotion in children with ASD, although recent infant fMRI work suggests that some infants at high-risk for ASD may present atypical neural responses to emotional (i.e., sad) vocalizations (Blasi et al., 2015). A first study has shown lower Mismatch negativity (MMN) amplitudes in response to angry but not happy voices in individuals with ASD, possibly reflecting atypical early sensory processing of negative emotion (Chin-hsuan, 2011). A second study examined the electrophysiological correlates of vocal emotion processing in 10–16-years-old with ASD using an emotion recognition task. Stimuli consisted of the phrase “I’m leaving the room now, but I’ll be back later” spoken in happy, angry, sad and fearful tone of voice (Lerner et al., 2013). This study found a significant correlation between emotion recognition errors and N100 latency, suggesting that participants with longer N100 latencies made more recognition errors. The findings were interpreted as indicating difficulties with speed of sensory processing of social information in ASD as reflected by N100 latency (Lerner et al., 2013). An important limitation of this study, however, was that it lacked a group of typically developing children to inform our understanding of the degree of abnormality of this processing. Similar work has investigated the neural correlates of vocal anger processing in 14 9–12-years-old boys with Asperger syndrome (AS) and 13 controls using a passive oddball paradigm (Korpilahti et al., 2007). Vocal stimuli consisted of the word ‘*Anna!*’ spoken with tender and angry tone of voice. Although the study did not report a differential neural response to emotion condition in any group, the N100 component peaked later in children with AS compared to controls (Korpilahti et al., 2007).

In summary, findings provide some evidence that impaired automatic discrimination of facial and vocal expressions may be a candidate neural marker of the social impairments observed in ASD. A limitation in existing research using vocal stimuli is that they include semantic or lexical confounds in the tasks. This raises the possibility that findings are influenced by differences in language comprehension (Paul et al., 2005). Future studies should employ pure prosodic stimuli devoid of semantic or lexical content.

### Implications for Early Detection and Preventive Intervention

Future research should aim to elucidate the sensory, perceptual and cognitive processes (i.e., mechanisms) underlying emotion processing. Emotion-specific neural markers can be useful in identifying individuals most in need of preventive intervention as well as identifying risk and resilience factors for disorder-specific outcomes. Targeted prevention programs can help children read emotions in others successfully or help implement strategies to compensate for emotion-related abnormalities. This can help reduce the risk for later emerging psychopathology. It is critical to intervene early in order to prevent a number of problems before they manifest and help reduce the economic and social burden of mental disorders for individuals and society.

## Author Contribution

The author confirms being the sole contributor of this work and approved it for publication.

## Conflict of Interest Statement

The author declares that the research was conducted in the absence of any commercial or financial relationships that could be construed as a potential conflict of interest.
